# Full title: “Hopes, worries and expectations” experiences of pregnancy with inflammatory bowel disease: An interpretative phenomenological analysis study

**DOI:** 10.1016/j.heliyon.2024.e31954

**Published:** 2024-05-25

**Authors:** Rebecca Homer-Perry, Wladyslawa Czuber-Dochan, Tiffany Wade, Satvinder Purewal, Sarah CE. Chapman, Matthew Brookes, Christian P. Selinger, Helen Steed

**Affiliations:** a University of Wolverhampton, Faculty of Education, Health, and Wellbeing, Psychology Department, Wulfruna Street, Wolverhampton, WV1 1LY, United Kingdom; b Florence Nightingale Faculty of Nursing, Midwifery and Palliative Care, King's College London, United Kingdom; c Department of Pharmacy & Pharmacology, Faculty of Science, University of Bath, United Kingdom & Institute of Pharmaceutical Science, King's College London, United Kingdom; d Gastroenterology Unit, New Cross Hospital, Wednesfield Road, The Royal Wolverhampton NHS Trust, United Kingdom; e Gastroenterology, Leeds Teaching Hospitals NHS Trust, St James' University Hospital, Leeds, United Kingdom

**Keywords:** Inflammatory bowel disease, Pregnancy, Reproductive health

## Abstract

**Background and Aims:**

Inflammatory Bowel Disease (IBD) affects many women of childbearing age. High levels of voluntary childlessness and high levels of pregnancy-related fears have been reported amongst these patients in several quantitative studies. We investigated the lived experiences of pregnant patients to better understand decision-making processes around family planning.

**Methods:**

Nine participants between 7 and 34 weeks pregnant (6 Crohn's Disease/3 Ulcerative Colitis), with an age range of 22–39 were recruited prospectively from three United Kingdom hospitals. Semi-structured interviews were conducted, and audio recorded. Interpretative phenomenological analysis was used to interpret the data.

**Results:**

Two main themes emerged: 1) IBD is perceived as a threat to family planning; and 2) healthcare professional advice, support, and reassurance was important. IBD was viewed as a potential threat to fertility and reproductive health. Consequently, women's lived experience of pregnancy is shaped by anxiety and pregnancy-related worries for mother and baby. Mothers actively sought out expert medical assurances to alleviate some of the perceived fears.

**Conclusion:**

Previous research has repeatedly found that women with IBD exhibit high levels of pregnancy-related worries and anxieties. Our findings find that high levels of anxiety are due to patients’ perceptions that IBD is a threat to their reproductive health and their offspring. Women relied on a medicalized discourse to understand their IBD experiences during pregnancy and actively sought biomedical resources for assistance before and during pregnancy. Consultants should be aware that when dealing with pregnant patients, some women may experience anxiety and require extra support.

## 1 Introduction

Inflammatory bowel disease (IBD) is a relapsing and remitting condition that includes Crohn's disease (CD) and ulcerative colitis (UC). IBD prevalence is increasing, and new diagnoses peak in child-bearing years [1]. Pregnancy outcomes for healthy IBD patients with inactive disease are largely comparable to the general population [2,3]. Exacerbation of disease, however, is usually linked to an increased risk of preterm delivery and low birth weight [3,4]. Furthermore, IBD patients have reported high levels of pregnancy-related fears and anxieties, including worry about IBD impacting pregnancy and the fear of disease transmission to their child [5,6]. In a systematic review, our group [7] found that IBD patients reported anxiety regarding medication, and up to half of IBD patients had insufficient knowledge of the implications of IBD on pregnancy and fertility [8]. Research [7] also revealed that few studies had explored the anxieties of women in depth around pregnancy to identify their specific concerns, how they managed these concerns, and their experiences and needs for support.

Several questionnaire-based studies have revealed apprehension about the consequences of IBD on mothers and babies [[9], [10], [11], [12]]. However, most of these studies have relied on closed-ended and pre-determined questionnaires and may have missed important, contextual explanations underpinning women's anxieties and worries. Limited qualitative studies have also found consistent evidence that women with IBD are concerned about the potential negative effects of their medication on their pregnancy and baby [13,14]. Previous research highlighted specific medication-related concerns, such as a perceived negative impact on a child's immune system and a lack of information [13]. Parallels between IBD and motherhood emphasized the significance of preparation, lifestyle modifications, and monitoring personal and physical development [14]. The systematic review [7] informed the rationale, to investigate the anxieties women with IBD experience during pregnancy. There is a lack of understanding of the lived experiences of pregnancy for women with IBD and a lack of in-depth investigation into IBD participants' experiential narratives/reflections on pregnancy experiences. The current study aims to provide a contextual understanding of the lived experience of pregnancy for women with IBD, while looking at how pregnancy and IBD information can be integrated into routine IBD care to better prepare women for pregnancy.

## 2 Method

### 2.1 Design

Semi-structured interviews using Smith, Flowers, and Larkin's [15] Interpretative Phenomenological Analysis (IPA) were used.

### 2.2 Recruitment

Purposive sampling was used to recruit women that meet the criteria, which were being over the age of 18, living with Crohn's or Colitis, and were currently pregnant. They were recruited from a combination of IBD clinics, from 3 United Kingdom centers in geographically distinct areas (Leeds, London, Wolverhampton), and via social media with the support of Crohn's and Colitis UK. One of the recruiting clinics was an IBD pregnancy clinic. Participants were excluded if they had assisted conception, patients with specific language requirements due to interpretation facilities being unavailable or they had a certain type of disease such as microscopic colitis, therefore only UC and CD were included as they are the disease type associated with risk of adverse pregnancy outcomes.

### 2.3 Data collection

The study was developed in collaboration with a dedicated Patient and Public Involvement (PPI) group comprised of women with IBD. We further sought PPI comments at a ‘National IBD Patient Involvement in Research Day’ organized by Crohn's and Colitis UK (2018). Patient engagement informed the study design, topic guide, (and preference for interview mode (face-to-face or online). A topic guide for semi-structured interviews was informed by a systematic review conducted by our research team [7].

Eligible participants were approached by either IBD nurses or the research team and provided with a detailed information pack of the study and invited to participate. Interested participants were provided with opportunities to ask questions and get further information and asked to provide informed consent. Participant's preferences for phone/person interviews and the gender of the interviewer were also adhered to. Two female researchers (WCD or RH-P) carried out the interviews via telephone (n = 7) and face-to-face (n = 2). All interviews were audio-recorded and took place between December 2018 and November 2019, lasting up to 60 min, totaling 5 h and 27 min.

In accordance with IPA and the methodological requirement of homogeneous samples, this data set focused only on the pregnant participants - who are a subset of the whole sample (n = 23 Female IBD patients and n = 4 partners). The inclusion and exclusion criteria were the same for the whole data set, which also includes patients that were either actively family planning, post-partum or voluntarily childless. The only criterion for the partners was they had to be in a relationship with someone that has IBD and they had no specific language requirements. These other group data sets will be reported separately in further papers.

### 2.4 Data analysis

All interviews were transcribed verbatim by a professional transcriber. Data were analyzed utilizing excel and following Smith, Flowers, and Larkin's [15] underlying methodological principles and steps for IPA (see Table 1 for a breakdown of the six stages of IPA). We were able to incorporate participants' narratives and interpreted meaning into first-person experience-based structures of consciousness by utilizing these methods. The first author's interpretation and analyses were discussed between RH-P, SP and HS and we tested the findings against data samples and different potential viewpoints. Our themes were generated for the whole group, which represented a conceptual structure that reflected the phenomenon, expressing its shared and underlying psychological meaning.

**Table 1:** Process of data analysis using Interpretative Phenomenological Analysis (IPA) [15].

Stage	Activity	Action
**1**	Read and re-read transcripts to get to know the data	The researcher read and re-read every transcript to become familiar with the data and the topics covered. This was done to gain familiarity with the data. The participants received pseudonyms, and the transcripts were made anonymous. Names of people or organizations mentioned throughout the interview were changed to reflect their professional or social position, such as “gastroenterologist,” “IBD nurse,” “hospital,” “wife,” “husband,” “baby” etc.
**2**	Initial notes to systematically capture observations	Significant responses from the reader, statements, sentences, or quotes are identified in the transcript. Initial notes and exploratory comments were made.
**3**	Develop Emergent Themes	Identification of and labelling of major and minor themes.
**4**	Connection Across Emergent Themes	Clusters of themes are labelled in a way that captures their essence.
**5**	Next Case	Stages 1–5
**6**	Patterns Across Cases	Once analysis was completed on individual transcripts (steps 1–5), the subordinate themes were analyzed across participants (step six) to form the themes and sub-themes for the group. This includes searching for connections across participants, developing recurrent group themes and subthemes. Abandoning or combining underrepresented themes.

### 2.5 Ethical considerations

The study received ethical approval by North-West Preston REC, reference: 18/NW/0361 (July 2018). Ethical considerations were considered throughout the conduct of this research and have helped to ensure that the needs and rights of both participants and the researchers have been respected and protected. The research and its procedures have abided by the relevant policies of the Integrated Research Application System (IRAS) ethics committee and adhere to the British Psychological Society (BPS) codes of ethical conduct.

## 3 Results

### 3.1 Participants demography

Thirteen pregnant participants were approached, two withdrew and two were ineligible. Nine participants were interviewed (mean age = 31 years (range 22–39 years) who were between seven and 34 weeks pregnant. Eight participants identified as White/British; one was White/Any; five were married; and four shared a home with a partner. Eight participants were expecting their first child; three women had previously miscarried. One was in her first trimester, five were in their second trimester, and three were in their third trimester (see Table 2).

**Table 2:** Demographics of pregnant IBD participants.

Participant pseudonym names	Age (years)	Crohn's disease (CD)/Ulcerative Colitis (UC)	Length since diagnosis	Last time you experienced IBD symptoms	Weeks pregnant	Reproductive history	Relationship status	Medication
Jessica	33	UC	13 years	13 years	24	Primigravida	M	Adalimuman other 5ASA
Tracey	36	CD	7 years	3 years	28	Gravida 3, para 1	M	Pentasta 3g od years. 6mp 50 mg years
Daisy	22	CD	5 years	6 months	34	Primigravida	LWP	Azathioprine 100 mg a day, B12 injections every 7 weeks and iron tablets
Poppy	31	UC	6 years	7 months	20	Primigravida	M	Pentasa 2 mg twice a day and Enema 3 times a week
Lynn	39	CD	4 years	4 years	17	Gravida 3, para 0	LWP	Pentasa 2 mg once a day
Dawn	34	UC	5 years	6 months	30	Gravida 2, para 0	LWP	mezavant XL 2 tablets a day
Rose	29	CD	5 years	3 months	16	Primigravida	M	Azathioprine 75 mg od 2014 & Infliximab 10 mg/kg 8 weekly 2016
Sarah	29	CD	10 years	2 days	17	Primigravida	LWP	Humira 40 mg pen, every 2 weeks
Tina	28	CD	15 years	2 years	7	Primigravida	M	Octasa and folic acid

M = married, LWP = living with partner.

### 3.2 IPA results

IPA highlighted two interconnected features of women's understanding of IBD during pregnancy (see Table 3). Both elements related to how women used medicalized communication to understand their lived experiences with IBD and pregnancy.

**Table 3:** Themes and sub-themes.

Themes	*IBD is perceived as a threat to family planning*	*Number of participants indicated themes*	*Need for Health Care Professional's (HCP) reassurance.*	*Number of participants indicated themes*
Subthemes	Threat to fertility	8/9	Support sought from Health Care Professionals (HCPs)	8/9
	Worries for mother and baby's health	7/9	Self-searched resources	6/9
	Worries about IBD treatment	8/9		

First, IBD was presented as a threat to women's reproductive health, leading to anxiety and pregnancy-related concerns for mother and baby to influence experiences of pregnancy. Secondly, the mothers actively sought Health Care Professional (HCP) assistance (including HCP consultations, medical pamphlets, and online resources) to assuage their anxiety.

## 4 IBD is perceived as a threat to family planning

### 4.1 Threat to fertility

Eight out of nine women framed IBD as a physiological threat to family planning – IBD was seen as a potential physical and medical disruptor in their ability to become pregnant and have a healthy pregnancy. IBD symptoms were a particular concern during conception and pregnancy, with wide variations in concerns on whether they improved, worsened, or remained stable.

IBD diagnosis for some women was an anxious period. Participants described considering their fertility differently in response to their IBD diagnosis depending on their age and where they were in their life (e.g., at diagnosis two were teenagers, and two were in their early twenties and were not considering family planning). Advice was sought about fertility by five participants at diagnosis, even though they were not considering starting a family at the time.

‘When I was diagnosed, the first thing and the only thing I asked was if that was going to affect having children’ (Tracey, CD, 36 years old, age 29 at diagnosis)

Seven participants reported initial concerns that IBD could affect their fertility. Conversely, two participants did not report any particular concern about the impact of IBD on their reproductive health. Several participants also discussed having to time their pregnancy ‘right’ with IBD, reporting either trying to conceive immediately during a period of remission (due to fears of a disease flare derailing their pregnancy) or having to postpone their plans for pregnancy due to active IBD. For example, one of the participants mentioned that a flare-up had caused feelings of helplessness, and she demonstrated the urgency and threat that IBD brought to her family planning decisions during this narrative:

‘I think it became apparent to me that it was a kind of now or never, that if I didn’t conceive now, the likelihood of me being able to later down the line might be harder, purely because my disease might get worse, so I think for me it was yes, it was a little bit of a gun pointed towards my head.’ (Jessica, UC, 33 years old, age 20 at diagnosis)

The use of ‘*gun pointed towards my head’* metaphor particularly highlighted the perceived threat and lack of choice in timing for pregnancy and sense of urgency. The participants ideally wanted what they perceived to be a ‘normal’ experience, which is, to be able to fall pregnant easily and have a trouble-free pregnancy and to not worry about their own health or the health of their baby. IBD was perceived as putting the participants at a disadvantage in terms of being ready to meet the ‘normal’ challenges of life, including the hope of becoming a mother.

### 4.2 Worries for mother and baby's health

Participants reported worries regarding maternal and neonatal outcomes. Although their IBD may have changed the participant's trajectory of a planned pregnancy, they did not deviate from their desire of wanting a family. Participants had to constantly reconcile their wish to become a mother with their fear of the negative effects of IBD on their and their child's health.

‘I didn’t want to be pregnant when I was having a flare up, but it didn’t stop me from wanting to become pregnant’ (Poppy, UC, 31 years old, age 25 at diagnosis)

For some participants, worries about fertility were followed by anxiety and worries throughout pregnancy regarding adverse pregnancy outcomes. Not all women had anxiety before becoming pregnant; two participants had been in remission for over a year and thought there was no reason to worry. Others were concerned about being admitted to the hospital due to pregnancy-related flare-ups. Because of the unpredictability of their disease, they felt a loss of control, which increased worries about themselves and the baby. A common word in participants' narratives was ‘fear’ - they feared a flare-up during pregnancy and/or birth.

‘It’s always a bit of a shadow over your shoulder at any point, and I do always have that awareness in the back of my mind, that it could kind of flare up and I guess that was a bit of the pregnancy, is that the time it’s going to do that’ (Tina, CD, 28 years old, age 13 at diagnosis)

These fears influenced participants' behaviour. For Tina, IBD is described as a ‘shadow’, a dark presence over her ‘shoulder’ – which suggests it's a burden to bear. Due to a sense of ‘fear’ for their health, one participant avoided contact with small children or sick adults due to the risk of contracting viruses. Other participants experienced periods of social isolation due to IBD symptoms, such as fatigue.

‘Obviously I have flu vaccinations every year but I think I’ve kept myself to myself quite a bit during my pregnancy, kept away from small children, kept away from people that I know have been sick. I had a very good friend who had pneumonia, I very much kept away from her for quite a while, simply because I couldn’t afford to risk getting any, contracting anything so purely because I knew that my immune system was probably a little bit more, a little bit lower than the average person’ (Jessica, UC, 33 years old, age 20 at diagnosis)

In this extract, Jessica highlighted how she drew on a self-reliance discourse both to find solutions to her problem and emotionally. Keeping herself to herself is a social practice that reflects Jessica's acknowledgment of her IBD during pregnancy, maintaining a degree of control.

Fears for the baby were very common too. Some of the participant's expressed a desire to protect the unborn baby. One worry voiced by four participants was their baby inheriting IBD.

‘The one problem that I did have was that I was worried that the baby might potentially get Crohn’s. That’s the kind of concern that I have’ (Daisy, CD, 22 years old, age 17 at diagnosis)

Another patient was concerned if she and her partner both have IBD, would their child inherit IBD? However, she reconciled her fears for the inheritability of IBD and her desire for a child:

‘I think it’s really valuable to recognize that if baby turns out to have IBD, we’re still alive, baby will still be alive and you can get through it, you just deal with things as they come’ (Rose, CD, 29 years old, age 24 at diagnosis)

Rose prioritized the baby's life with a positive attitude to an unknown outcome. This approach *‘you just deal with it’* was embedded in a deep sense of responsibility for the fetus. Five participants reported the first trimester as particularly stressful because of uncertainty and difficulty balancing worry and excitement:

‘I was obviously excited but worried, just generally worried about the pregnancy in the first 12 weeks and what might happen’ (Poppy, UC, 31 years old, age 25 at diagnosis)

During pregnancy, worries and anxieties shifted towards the baby's health. Six participants conveyed persistent uneasiness relating to their IBD during pregnancy. Participants who had previously experienced a miscarriage worried their illness caused the miscarriage.

‘I wouldn’t say it’s the most enjoyable time … as I suppose the digestive problems that I get makes things a bit worse. And then having had a miscarriage obviously the first few weeks were always a bit of a worry that something’s going to go wrong again.’ (Dawn, UC, 34 years old, age 29 at diagnosis)

These participants were concerned about further risk of miscarriage, particularly the three that had previously miscarried, but two other participants worried about this as well. They did not trust their own bodies, heightening worries about adverse outcomes:

‘*You read up on, if you’ve got Crohn’s, you can have a small birth weight baby, premature baby, stillborn baby’ (Lynn, CD, 39 years old, age 35 at diagnosis)*

### 4.3 Worries about IBD treatment

IBD treatment (medication and surgery) was a further source of anxiety and deliberation in pregnancy experiences and choices. Participants discussed whether they should continue medication or stop taking their medication, and implications for them and their baby. These dilemmas shaped some of their decisions:

‘I just thought I was being ignorant, I just thought I could, I was a superwoman and I thought I could you know, come off it [medication] for a couple of weeks and I was fine ….‘I’d stopped taking the medication and the IBD nurse said no, don’t stop taking medication, you’re on this for life now.’ … ‘I just thought I’d been cured but obviously there’s no cure.’ (Lynn, CD, 39 years old, age 35 at diagnosis)

The ‘superwoman’ idea is a fantasy of good health, strength, and boldness. Similar to participants' comparisons to ‘normal’ pregnancy, perceived as problem-free and easy, Lynn's desire to be ‘superwoman’ and medication-free was not aligned with her reality.

Women expressed concerns about the effects of medications and how infections may affect their pregnancy:

‘*More to do with the drugs, I think. I think you’re very conscious that because you are potentially immunosuppressed yourself that getting a cold or getting the flu or something like that can have a bigger impact on you than it would do on your average pregnant person who doesn’t have any health issues.’ (Jessica, UC, 33 years old, age 20 at diagnosis*)

There were dilemmas over the impact of medication, particularly biologic medication (e.g. Adalimumab and Infliximab). On one hand, the participants were concerned that stopping their medication may lead to flare-ups. But they were worried about the impact of these medications on the unborn baby. These narratives are like previous narratives of reconciling desire for children with worries about child's health. In this case, the medication was viewed as a necessity, and yet, there was great concern about the impact the medication could have on the mother and baby.

‘I asked for some advice about my medication … the baby obviously … gets what I get, would she be on the drug that I’m on, and would she get poorly while coming off it, and would I need to be extra careful with anything’ (Daisy, CD, 22 years old, age 17 at diagnosis)

Daisy and the other two participants were more concerned about taking their medication to ensure they are healthy and therefore the baby is healthy. Some of the worries related to medication and its impact on healthy conception, their mental health, maternal health, and the baby's health. Five participants communicated that they took supplemental vitamins and minerals, such as Vitamin D and folic acid to support their pregnancy. There was uncertainty amongst some of the participants with the impact/benefit of taking these supplements, due to IBD being exacerbated at times.

## 5 Need for Health Care Professional's (HCP) reassurance

### 5.1 Support sought from Health Care Professionals (HCPs)

Participants draw heavily on a medicalized discourse in their meaning-making of the impact of IBD on family planning and perceived IBD as a threat to their family planning, and the health of mother and child, which caused worries and anxieties. Consequently, participants actively sought reassurances from HCPs to help alleviate their anxieties. Participants who sought advice from their HCP took more ownership over their family planning choices and understanding of their experiences. First, participants used their HCPs for knowledge and information, and for reassurance for their pregnancy. For many participants, the HCPs were perceived to be valuable resources and potentially provided participants with some reassurance and certainty against the uncertain nature of IBD disease and their pregnancy related worries. ‘Reassurance’ was a word often used by participants to describe either what they wanted from their HCPs or what their HCPs provided.

‘I didn’t trust the GP an awful lot because I know they don’t specialize in it [IBD] … I’m really confident in what he [the consultant] tells me and I see him all the time now, I don’t see any other doctors'. (Poppy, UC, 31 years old, age 25 at diagnosis)

Familiarity with their HCP was also important to participants as it seems the HCPs are looking after the mother-to-be and the baby, whereas the participants appeared to be more focused on the baby first and themselves second. Many of the participants praised their IBD nurses for their care. Specifically, they emphasized that the nurses were the ‘constant faces' with whom they formed relationships, whereas consultants are frequently different, making participants feel as if they must re-tell their story/history in each consultation. Eight participants mentioned having access to the IBD helpline, which is handled by the nurses and where they may email or call and receive a response in a ‘reasonable period’ of 24–48 h. As a result, having a recognizable face among the HCPs team offered participants confidence and reassurance.

‘If I have got any problems, I’ve got the helpdesk you know, the helpline I can call them and yes, I feel pretty supported in that instance.’ (Tracey, CD, 36 years old, age 29 at diagnosis)

IBD nurses were seen as direct care providers, the initial point of contact for participants, educators, biologics administrators, and social support providers. One participant stated ‘they really know their stuff’ therefore leaving the women reassured that they do not necessarily have to wait for an appointment with the consultant. IBD participants pregnancy management means they receive additional neonatal scans and tests and many participants responded positively to these additional tests. Having these additional appointments appeared to please some participants because they felt that their HCPs were keeping on top of it.

‘I think they’ve been pretty reassuring yes … sometimes you just want to be reassured even more and like just to double check’ (Sarah, CD, 29 years old, age 19 at diagnosis)

It seems Sarah is regaining control by wanting to make sure everything is how it should be, ‘double-checking’ even after being reassured, demonstrating the emotions of uncertainty participants face due to the complexities in their pregnancy due to IBD.

Although all participants perceived ultrasound scans as reassuring, the period of waiting before and between scans was a nervous time, potentially heightening their anxiety. One of the patients was first concerned about the increased frequency of monitoring the baby, but she now finds it reassuring, especially after a miscarriage. However, the reassurance from a scan appears to be temporary. Other patients had experienced similar, that the scans can reassure them and put their mind at rest nevertheless, it appears that they can not shake the worry about the effect the medication could be having on the baby, regardless of the reassurance:

‘I mean I know obviously they [scans] reassured me it will be fine, but you know, there’s always that kind of worry in the back of your head’ (Sarah, CD, 29 years old, age 19 at diagnosis)

Participants also appeared to value support more from their IBD HCPs than non-IBD specific obstetric and midwife HCPs. Some participants had little expectations from their midwives because they would be unable to answer specific questions relating to IBD. Further, some participants seemed disappointed that midwives did not ask any questions/details about their IBD, thus they relied more on their IBD team for pregnancy support.

‘Maybe if she’d [midwife] had a bit more awareness of the impact rather than just form filling, that could have been good … I have extra needs … I need full confidence, that’s just what I need’ (Tina, CD, 28 years old, age 13 at diagnosis)

Due to the midwife not meeting Tina's expectations of what Tina claimed to be “essential documents,” she felt that she could only have a limited amount of confidence in her. Because the midwife was portrayed as blasé, the patient felt she was not receiving the support and knowledge she needs as a first-time mother-to-be.

### 5.2 Self-searched resources

Participants did not rely wholly on their HCPs for information and many of them engaged in information-seeking behaviour about IBD and pregnancy. Generally, the HCPs were seen as the important source of information, however, participants searched for further information, mostly through the internet and health leaflets, as confirmation. The internet was perceived as the most accessible modality for participants to access current health information. Participants expressed that they evaluated the sources of the information, for example other patients can be trustworthy, but also inaccurate. Some chose websites that were seen as anxiety-free, such as those for medical journals or IBD-specific organizations, such as Crohn's and Colitis UK. Some used Google and various social media platforms, which were described as both “useful” and “worrisome”. Participants preferred a variety of sources for their information, including what was perceived as reliable:

‘I try my best to kind of look at the more you know, like scientifically accredited type documents as opposed to just kind of looking on random websites.’ (Sarah, CD, 29 years old, age 19 at diagnosis)

Although they did not always understand medical terminology, some participants used research papers which were perceived as legitimate sources, saying they felt more confident knowing it came from a specialist subject field. Although social media sites were not perceived as reliable, participants will still use them to ask questions about other patients' experiences with IBD and pregnancy. This demonstrated a need for emotive support and reassurance.

‘I think because I use the Facebook group daily, I use them, I sort of dip in and out throughout the day. And it was knowing other people’s experiences and that it was possible, and it wasn’t necessarily the end of the world. When I actually rang my GP to say yes I’m pregnant, he said would you like to continue, and it didn’t occur to me not to. And I think because, that was a lot of that was because of the support I get online.’ (Rose, CD, 29 years old, age 24 at diagnosis)

Rose appears to be taken by surprise by her GP's questioning on whether she would like to continue with her pregnancy. She attributes this to the support she gets from her online group and also learning from other women's similar experiences.

Participants were looking for information about the disease's nature, causes, medication, pregnancy, and pregnancy outcomes. However, not all experiences or answers were available online or perceived positively. For example, some participants' anxieties were heightened when they had concerns and could not find answers online. Conversely, when searches expanded their knowledge, some internet resources led participants to compare themselves to others and triggering additional questions than when they initially started looking. ‘Fear’ was one of the emotional responses caused by the overwhelming volume of online resources.

‘I’m very much someone who looks at other resources, but in this instance, I’ve let myself be guided by the different medical teams rather than putting the fear of god into myself by looking through countless, you know, online resources' (Jessica, UC, 33 years old, age 20 at diagnosis)

The breadth of information available caused a great deal of uncertainty; Jessica expressed frustration and dejection with online resources, acknowledging information can be misleading. Other participants were more optimistic, describing how looking at solutions, with a ‘positive’ attitude allowed them to avoid the negative aspects of the internet and utilize professional websites. Participants used the internet to discover both IBD-specific material, and pregnancy-related information, as well as answers to specific queries or reassurance.

## 6 Discussion

This is a collaborated study with IBD patients and public involvement, and one of the first 10.13039/100010827IPA studies examining the lived experiences of women with IBD whilst they are pregnant and highlights two main themes relating to 1) IBD is perceived as a potential threat to family planning, and 2) patients sought healthcare professionals' advice, support and reassurance. Women's experiences were complex and multi-layered, and all participants engaged in medical reassurance, which shaped their pregnancy experiences.

Many previous studies have consistently found women with IBD experience pregnancy-related fears and anxieties [9,10,16] including a limited number of qualitative studies [13,14]. Our findings demonstrated patients have high levels of pregnancy-related anxiety due to the perception that IBD threatens their reproductive health in wide-ranging ways [5,6]. These included fears for the baby's health, the possibility of passing on IBD to their child, and concerns over medical treatment during pregnancy. Patients prioritized concerns over the baby's health over their own e.g. when considering medication and nutrition fitting with other work that has highlighted concepts such as ‘self-sacrificing’ mothers [17], placing unborn babies' needs ahead of their own. This is consistent with prior research that shows IBD patients were most worried about outcomes for the baby such as miscarriage and whether this might occur as a result of the mother's IBD [3,4,7]. Medication was one of the topics that the women talked about the most since they were concerned about the effects it might have on the baby as well as the effects of not taking it on themselves. However, feelings towards medication were complex because many patients also acknowledged the need for medication to stay healthy during pregnancy. Other studies have also found that patients are concerned about the side effects of IBD medications [12,13,18]. Previous research found patients with less disease-specific pregnancy knowledge thought medications should be stopped prior to conception and pregnant women should stay away from all IBD medications [8].

Pregnancy-related worries meant women wanted reassurance – either from their HCPs and/or search for information. In previous research [19], patients with IBD valued advice from their doctor despite most also using the internet to look up medical information. Participants in our study also required more education and reassurance on medications [20]. While some participants acknowledged that non-IBD specialists (such as midwives, GPs, and obstetricians) could not be expected to have in-depth knowledge of IBD, participants nonetheless expressed a desire for the midwives to be more understanding of their unique needs due to IBD. We note an interesting effect of additional growth scans in pregnancy providing temporary reassurances but, in our participants’ experience that effect seemed short-lived and anxieties continued shortly after the scan. As miscarriages are relatively common [21], psychological support should be tailored for IBD women who may have had a miscarriage and may be experiencing heightened levels of anxiety from the start of their pregnancy.

Participants stated that they evaluated sources of information and believed that other patients could be trustworthy but also inaccurate. This fits with previous research [22], which discovered that negative experiences shared on social media caused concern because people assumed they would have the same experience. However, most believed that information provided by charities (such as CCUK) can be trusted. Only a small number of participants used social media to seek out positive experiences and advice from persons they perceived to be ‘like them’ (i.e., pregnant with IBD). However, anxieties were heightened by the inconsistency of information obtained and many participants expressed a desire for expert-led, medically based, truthful information from reliable, certified sources, this is consistent with previous research [23].

This study found that the relationship between patients and HCPs was important. HCPs were regarded as a trustworthy source of assistance and information before and during pregnancy. However, many of the participants did not disclose family planning until they were already pregnant, suggesting IBD services should routinely provide pregnancy counselling prior to pregnancy [24]. HCPs should be aware that IBD mothers-to-be may be anxious, and that this anxiety stems from the perception that IBD is a threat to their pregnancy, and baby. According to earlier research [25,26], women and HCPs perceive pregnancy threats differently. For some of our participants, concerns about the potential threat of IBD to their reproductive health may begin immediately after diagnosis. It would be advantageous if HCPs managing female patients with newly diagnosed IBD to address concerns to help dispel their fears. Further, our findings suggest HCPs could also engage in various other activities which could improve women's experiences of pregnancy. We recommend that: newly diagnosed patients are informed about the impact of IBD on fertility and pregnancy and reassured that IBD does not pose a threat when they are in remission. HCPs should encourage patients to discuss and potentially prepare for their family planning during their consultations. Women actively seek HCP resources for prenatal support. HCPs should share medical leaflets about family planning with patients, particularly information from patient-preferred sources such as charities (e.g., CCUK). During pregnancy, HCPs should be aware that patients may simultaneously find the additional ultra-scans/consultant-led pregnancy consultation reassuring and worrying. Finally, it is important that HCPs reassure patients about medication safety during pregnancy and address any concerns patients may have.

## 7 Limitations and future work

This study recorded perspectives on preparing for motherhood while living with IBD and offered a novel contextual explanation for the nature of anxieties, concerns, and information-seeking linked to family planning and pregnancy. There is limited qualitative data on pregnancy concerns among IBD patients. This study discovered themes comparable to recent studies [13,14] but provides a more detailed explanation of the causes of pregnancy anxieties and health-seeking behaviour, as well as the need for reassurances.

The study included a small group (n = 9) of women who were pregnant and produced a wealth of information. Eight of the participants were past the first trimester but not yet getting ready for birth. The focus therefore could have moved from certain events, including morning sickness, Braxton hicks, concern over early scans, abortion, miscarriage, and birth preparation. Patients were mostly from three UK centers with clinicians interested in IBD and pregnancy, therefore may have received more knowledge and support than is typical. Our cohort was predominantly affected by CD and the study participants may have experienced more active disease than generally seen. Since the goal of IPA studies is to collect detailed information that represents the experiences of study participants, they frequently only examine small samples. All IBD patients were informed about the study, however nobody from other educational backgrounds or ethnic minority groups volunteered to participate.

For future research, a longitudinal approach would capture the evolving experiences of women with IBD from early pregnancy to the postpartum period. Consider the inclusion of a more diverse group of participants (ethnicity, geographical location, experiences of different HCP and different stages of pregnancy), as well as more even numbers of CD and UC patients.

## 8 Conclusion

Our findings show how women's pregnancy-related behaviour is influenced by their perception of their pregnancy as a threat to both mother and child. To understand their lived experiences of pregnancy, patients relied heavily on a medicalized discourse. The fact that patients are concerned about their own health but continue to prioritize the baby's health over theirs in terms of medication and nutrition are key features of this. Women actively sought HCP's resources for prenatal support. HCPs are regarded as trustworthy sources of assistance and information. However having access to HCPs and having been given information does not always alleviate patients worries, as research [13,14] found IBD women report high levels of pregnancy-related anxieties, such as worry about IBD impacting pregnancy [13], the fear of disease transmission to their child [14], medication [7], increased risk of preterm delivery and low birth weight [3,4] and insufficient knowledge of the implications of IBD on pregnancy and fertility [8]. Demonstrating that IBD patients perceive that they are at more risk than the general population. Therefore, HCPs should be more proactive rather than reactive in providing help, support and advice to expecting mothers. This study provides context for why patients with IBD express pregnancy-related anxieties and concerns.

## Summary

Concerns regarding pregnancy outcomes influence how women with IBD approach family planning. Those who are pregnant recognized the need for more information and support.

## Key message

- Previous research has found that women with IBD exhibit high levels of pregnancy-related worries and anxieties.
- According to our findings, high levels of anxiety are caused by patients' views that IBD is a threat to their reproductive health and babies.
- Consultants should be aware that some women may experience anxiety and may need additional (psychological or information and reassurance) support from them.

## Disclosure and conflict of interest

SP – received a research grant from Tillotts.

MJB - has received grants and travel expenses from Vifor International and Tillots Pharma, outside of the submitted work.

CPS – CPS has received unrestricted research grants from Janssen and AbbVie, has provided consultancy to Warner Chilcott, Dr Falk, AbbVie, Takeda, Fresenius Kabi and Janssen, and had speaker arrangements with Janssen, Dr Falk, AbbVie, Pfizer and Takeda.

HS–HS has received an unrestricted research grant from Tillotts and had speaker arrangements with Almirall.

## Data availability

The data that has been used is confidential

## Funding

Funding this work was supported by the 10.13039/501100003522Crohn's and Colitis UK ‘[grant number SP2017/1]’ Living with IBD Award 2017.

## CRediT authorship contribution statement

**Rebecca Homer-Perry:** Writing – review & editing, Writing – original draft, Methodology, Investigation, Formal analysis, Data curation, Conceptualization. **Wladyslawa Czuber-Dochan:** Writing – review & editing, Methodology, Investigation, Conceptualization. **Tiffany Wade:** Writing – review & editing, Methodology. **Satvinder Purewal:** Writing – review & editing, Writing – original draft, Methodology, Investigation, Funding acquisition, Data curation, Conceptualization. **Sarah CE. Chapman:** Writing – review & editing, Methodology, Formal analysis, Conceptualization. **Matthew Brookes:** Writing – review & editing, Methodology, Investigation, Conceptualization. **Christian P. Selinger:** Writing – review & editing, Methodology, Investigation, Conceptualization. **Helen Steed:** Writing – review & editing, Methodology, Investigation, Formal analysis, Conceptualization.

## Declaration of competing interest

The authors declare the following financial interests/personal relationships which may be considered as potential competing interests:Satvinder Purewal – received a research grant from Tillotts. Matthew J Brookes - has received grants and travel expenses from Vifor International and Tillots Pharma, outside of the submitted work. Christian P Selinger – has received unrestricted research grants from Janssen and 10.13039/100006483AbbVie, has provided consultancy to Warner Chilcott, Dr Falk, AbbVie, Takeda, Fresenius Kabi and Janssen, and had speaker arrangements with Janssen, Dr Falk, AbbVie, Pfizer and Takeda. Helen Steed – has received an unrestricted research grant from Tillotts and had speaker arrangements with Almirall.
