# Supplementary material for: Full title: “Hopes, worries and expectations” experiences of pregnancy with inflammatory bowel disease: An interpretative phenomenological analysis study
Source: Heliyon. 2024 May 25;10(11):e31954. doi: 10.1016/j.heliyon.2024.e31954 (PMC11167349; doi:10.1016/j.heliyon.2024.e31954)
Supplement: Multimedia component 1 [file mmc1.docx]

| **Opening question:** | **Can you tell me about your experience of having IBD (CD or UC) and if and how this affected your plans for becoming pregnant? (women)** |
| --- | --- |
| *Listen for and follow-up on any of these aspects:* | - *Tell me about your experience of being pregnant in context of your IBD.* - *At what age were you diagnosed with IBD? When did you start thinking about starting a family in relation to the time of being diagnosed with IBD? When did you meet your partner?* - *Do you have any concerns about being pregnant in the context of your IBD? Do you have any specific concerns related to IBD?* - *When did you start to plan a family? Was this pregnancy planned?* - *Do you think that IBD affects your relationship with your partner?* - *Has your pregnancy affected your IBD symptoms in any way?* - *How does your partner feel you being pregnant in context of your IBD? Does he/she have any concerns? Have you talked about it?* |
| **Probing questions:** | **Did you talk to health professionals about starting a family?** |
| *Listen for and follow-up on any of these aspects:* | - *Who did you talk to?* - *What information related to pregnancy did you ask for?* - *Did you receive all the information that you needed?* - *Who else did you talk to about starting a family? Other members of healthcare team (GPs, nurses, consultants)?* - *Was the information relevant and useful to help you in your decision about staring a family?* - *How did that make you feel?* |
| **Probing question:** | **What other sources did you get your information from about family planning in the context of IBD? ?** |
| *Listen for and follow-up on any of these aspects:* | - *Do you have any personal contacts with friends/family diagnosed with IBD who have a family or are planning a family?* - *Do you engage with a wider IBD community through e.g. social media / support groups for information about pregnancy and IBD?* - *What other sources have you accessed to get your information about pregnancy and IBD?* - What information and knowledge about pregnancy and IBD does your partner have? - *What support do you have?* |
| **Probing question:** | **Information or support about pregnancy in IBD** |
| *Listen for and follow-up on any of these aspects:* | - *What information and support has helped you with your decision about starting a family?* - *What information and support do you need now while you are pregnant?* - *Do you feel that you have access to help and support that you need?* - *Is there any other information and support that would help you now?* - *What advice would you give to others with IBD considering parenthood?* |
| **Closing question:** | **Is there anything else that you would like to add?** |

| **Opening question:** | **Can you tell me about your experience of your partner having IBD (CD or UC) and your plans for having a family? (partners)** |
| --- | --- |
| *Listen for and follow-up on any of these aspects:* | - *Tell me about your experience of your partner being pregnant.* - *Do you have IBD? If yes, when were you diagnosed? Do you have any concerns about starting a family with you and your partner having IBD? What are your concerns?* - *When did you and your partner start to plan to have a family?* - *What concerns do you have about your partner being pregnant in context of her IBD? Please tell me more about that.* - *Do you think your partner’s IBD (and your IBD, if they are also diagnosed) affects your relationship?* - *How do you feel about your partner being pregnant in context of her IBD?* - *Do you have any other concerns? Have you talked about it?* |
| **Probing questions:** | **Did you talk to health professionals about starting a family?** |
| *Listen for and follow-up on any of these aspects:* | - *Who did you talk to?* - *What information related to starting a family did you ask for?* - *Did you receive all the information that you needed?* - *If yes / no, how did that affect your decision about starting a family?* - *Who else did you talk to about starting a family? Other members of healthcare team (GPs, nurses, consultants)?* - *Was the information relevant and useful to help you in your decision about staring a family?* - *How did that make you feel?* |
| **Probing question:** | **What other sources do you get your information from about family planning in the context of IBD? ?** |
| *Listen for and follow-up on any of these aspects:* | - *Do you have any personal contacts with friends/family diagnosed with IBD who have a family or are planning a family?* - *Do you engage with a wider IBD community through e.g. social media / support groups for information about pregnancy and IBD?* - *What other sources have you accessed to get your information about pregnancy and IBD?* - *To what extend does your knowledge about pregnancy and IBD come directly from your partner?* - *Do you take active part in antenatal activities? How do you feel about it?* |
| **Probing question:** | **Information or support about pregnancy in IBD** |
| *Listen for and follow-up on any of these aspects:* | - *What information and support has helped you with your decision about starting a family?* - *What information and support do you need now while your partner is pregnant?* - *Do you feel that you and your partner have access to help and support that you need?* - *Is there anything else that you or your partner may need while she is pregnant?* |
| **Closing question:** | **Is there anything else that you would like to add?** |
